# The prognostic value of blood glucose level on admission in non-diabetic patients with acute myocardial infarction

**Published:** 2009

**Authors:** V Chioncel, D Mincu, M Anastasiu, C Sinescu

**Affiliations:** * “Bagdasar-Arsen” Clinical Emergency Hospital

**Keywords:** diabetes mellitus, acute myocardial infarction, risk stratification, admission blood glucose level, mortality at 30 days

## Abstract

The diabetic patients represent a population with a high risk of morbidity and mortality because of vascular complications. Out of them, all the patients with acute ST- elevation myocardial infarction have a more reserved prognostic than those without diabetes mellitus. Moreover, the patients with impaired glucose tolerance have a more severe evolution in case of a myocardial infarction.

**Aim:** We wondered about the progress of patients with myocardial infarction and high blood glucose levels in admittance who had not been previously diagnosed with diabetes mellitus.

**Materials and methods:** We took 128 patients (who did not have diabetes) with acute ST- elevation myocardial infarction and divided them into three groups, according to the blood glucose level in admittance (<140mg/dL, 140-200mg/dL and > 200mg/dL); we also analyzed a group of diabetics with acute myocardial infarction who were admitted in the same period in our clinic. We made a prospective analysis over a period of 30 days. We evaluated the mortality at 30 days (as primary objective), as well as the extent of the infarction and the change of the left ventricle systolic function (secondary objectives).

**Results:** Both mortality and the mass of myocardial necrosis grew relative to the blood glucose level in admittance; instead, the ejection fraction varied inversely to the initial blood glucose level.

**Conclusion:** The admittance blood glucose level represents a useful and available marker for the initial stratification of risks in patients with acute myocardial infarction, even in those undiagnosed with diabetes mellitus.

## Introduction

During the last 25 years the coronary reperfusion therapies (angioplasty or thrombolysis) have considerably improved the prognosis in patients with acute myocardial infarction. Although the rate of mortality in patients with acute ST- elevation myocardial infarction who were treated with thrombolytic therapy or angioplasty has been significantly reduced, this varies a lot between the subgroups of patients in accordance with the appreciated class of risk based on clinical, biological, electrocardiographic parameters etc. In fact, different risk scores have been introduced for the stratification of patients and identification of those who need a more aggressive treatment. 

**Fig. 1:**
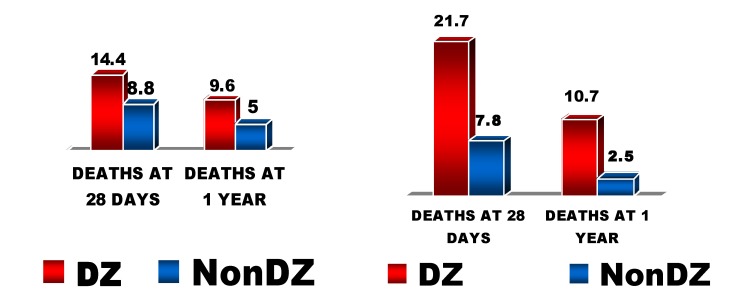
The mortality in diabetics with infarction varying with sex

Diabetes mellitus is a major health problem, characterized by a continuous growth of its prevalence worldwide. The cardiovascular pathology represents the most important cause of death in these patients. The patients with diabetes mellitus have a high prevalence of atherosclerosis and of coronary disease and have higher morbidity and mortality rates after myocardial infarction than those without diabetes mellitus. We also find a higher rate of complications of invasive coronary procedures (diagnostic or therapeutic) in these patients [**1**]. It is also known that mortality in patients with diabetes and myocardial infarction is significantly higher in women than in men [**2**] (**Fig. 1**).

However, the diabetic patients benefit less than non-diabetics from the active therapeutic principles based on evidence and used in the treatment of acute coronary syndromes [**3**]; thus, beta-blockers, in spite of evidence regarding the increase in survival (short term as well as long term) of patients with myocardial infarction, are >50% less utilized in patients with diabetes than in those without.

Therefore, there is a continuous concern regarding the finding of markers that characterize as accurately as possible the profile risk of the patients, as well as finding more appropriate therapies for them.

Various physiopathological mechanisms have been incriminated for the different behaviors of diabetics, such as: the exaggerate vascular inflammatory reaction (also shown by a higher growth of C-reactive protein in patients with diabetes), the increase of platelet aggregation and the stimulation of IIbIIIa glycoprotein and CD40 ligand molecule – factors that contribute to thrombus formation [**4**,**5**].

The patients with diabetes mellitus also have vascular tonus changes (due to endothelial dysfunction) that lead to impairment of the microcirculation and decrease of coronary flow; as a result, the “no reflow” phenomena after angioplasty occurs, and is more frequently found in patients with diabetes or hyperglycemia [**6**].

The patients with diabetes have increased levels of von Willebrand factor, which correlates with vascular complications [**7**]; a correlation between insulin resistance and increased concentration of von Willebrand factor has also been demonstrated [**8**].

Also characteristic in diabetics is hyperfibrinogenemia, which is predictive in cardiovascular complications [**9**-**11**].

**Fig. 2:**
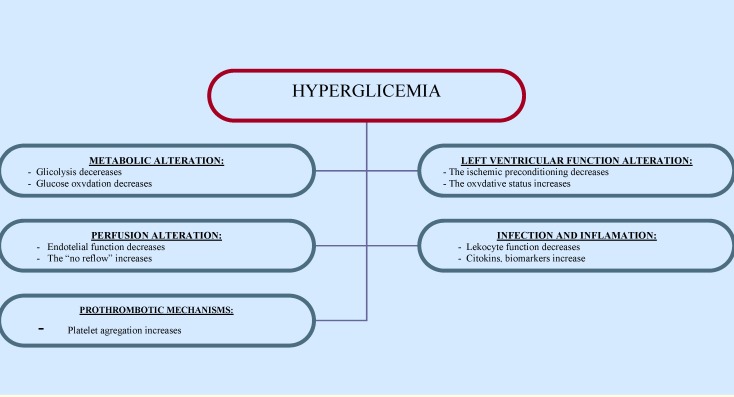
The physiopathologic consequences of hyperglycemia

Also, high levels of plasminogen activator inhibitor (PAI 1) and endothelia have been detected in the blood of patients with diabetes, as well as an increase in the activity of antithrombin 3, nitric oxide and prostacyclin – all these leading to the inhibition of fibrinolysis, acceleration of thrombus formation and impairment of coronary flux [**12**-**15**] (**Fig. 2**).

It is well known that the patients with impaired glucose tolerance (IGT) also have an increased incidence of cardiovascular complications [**16**].

Attempts have also been made to find a correlation between increased blood glucose values in admittance and intrahospital mortality of patients with acute myocardial infarction or those who underwent cardiopulmonary by-pass [**17**].

All these are physiopathologic reasons that explain the high rate of morbidity/mortality in patients with diabetes, being at the same time theoretical premises that can justify a difficult evolution in those with dysglycemia.

## The aim of the study

Beginning with the supposition of a dysglycemic status in patients with high blood glucose levels in admittance, we followed the association between the initial glucose level and short term mortality in non-diabetic patients with acute myocardial infarction.

## Materials and methods

128 patients with acute ST- elevation myocardial infarction have been included in the study, all of whom have been successively admitted in the Cardiology Clinic of “Bagdasar- Arseni” Emergency Hospital between 07.01.2006 and 06.30.2007.

The criteria for inclusion have been:

- angina > 30 minutes

- ST- elevation >0.1 mV in at least 2 contiguous derivations

- chest pain debut in the last maximum 12 hours in patients who were not diagnosed with diabetes

According to the blood glucose value in admittance the patients have been included in 3 groups:

- < 140mg/dL: 42 patients (group I)

- 140–200mg/dL: 49 patients (group II)

- > 200mg/dL: 37 patients (group III).

We have also monitored a group of 42 patients – known as diabetics, who had a myocardial infarction during the same period of time (07.01.2006 – 06.30.2007). 

The assessment of blood glucose levels was made in the hospital laboratory (the same one during the whole study) with normal limits established between 65-110 mg/dl.

**Fig. 3:**
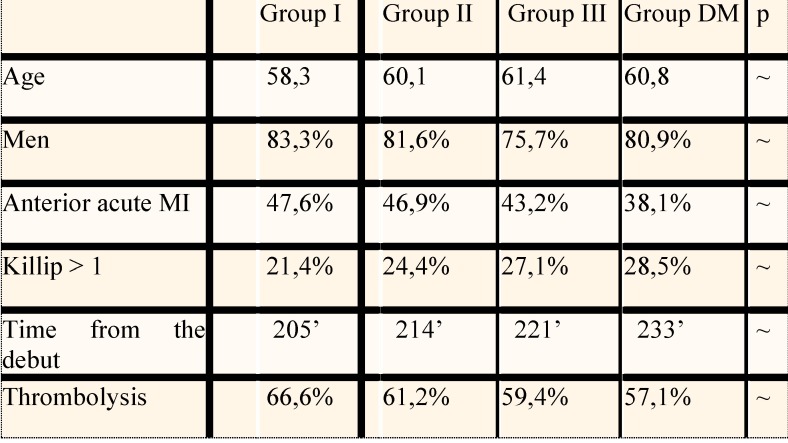
The structure of the groups included in the study

There were no noteworthy differences in the structure of the groups with respect to parameters such as: age, sex, localization of infarction, time from the pain onset, Killip class when they came in: most patients were men (~ 80%), the average age was ~ 60 years and the average time from the debut of infarction until admittance was of ~ 213 minutes. As clinical profile, most were in Killip I class, and the localization of infarction was almost evenly distributed between the anterior territory and the postero- inferior one (**Fig. 3**).

Approximately 62% of the patients received thrombolytic treatment – Streptokinase or Alteplase, according to the usual protocol: 

 - Streptokinase 1.500.000 U/30-60 minutes

 - Alteplase 100 mg/90 minutes 

 They also received the standard treatment for acute myocardial infarction:

 - An anticoagulant: Heparin iv 1000 U bolus, then 750-1250 U/h, for 2-5 days (~79% of patients) or Enoxaparin sc 1 mg/kg/day – 3- 6 days (~21%),

- An antiagregant: Aspirin 75-250 mg/day (~94% of patients) and/or Clopidogrel 75 mg/day (~63%), 

- A β-blocker (~63% of patients),

- A statin (~84%),

- An ACE inhibitor (~74% of patients) etc.

There were no patients who benefited from primary angioplasty (due to the lack of an available interventional cardiology laboratory) or antiagregant of the GP IIb/IIIa receptor inhibitors type.

 The treatment of the initial hyperglycemic values was the option of the physician on call, who chose one of the 3 following alternatives:

- insulin therapy – continuous iv perfusion (0.5-2 U/h, adjusted according to the blood glucose level) in 68 patients or intermittent administration of insulin in 13 patients

- oral antidiabetics – 38 patients

- combined therapy (insulin and oral antidiabetics) in 9 patients.

 As secondary objectives we also monitored: 

- the systolic performance of the left ventricle, expressed by the ejection fraction which was echographically evaluated (cardiac echography done in day 6-9 from admittance).

- the extension of the myocardial infarction – assessed enzymatically by the maximum LDH level (LDH ingathered daily until day 10). 

The statistics were made using the Ҳ² test, and statistically valid were the values < 0.05.

## Results

New data were gathered from all 170 patients included in the study (128 without known diabetes mellitus and 42 diabetics).

**Fig. 4:**
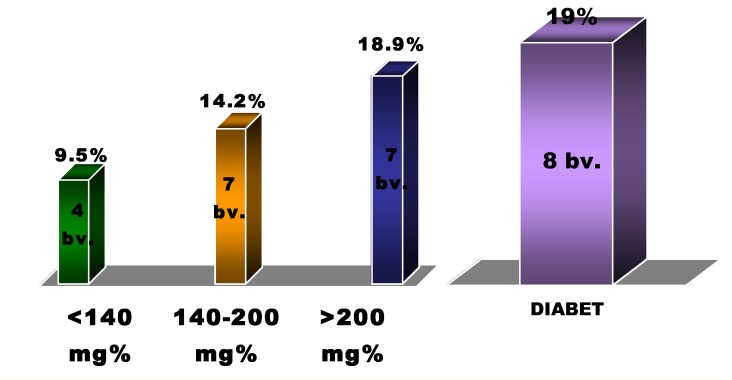
The mortality at 30 days in accordance with initial blood glucose level

Regarding the mortality within 30 days, progressive increase was noticed according to the value of blood glucose level; as such, those with admittance blood glucose level > 200mg/dL had a double mortality with respect to those with an initial blood glucose level below 140mg/dL (18,9% vs. 9.5%), the prognosis of the first being similar to that of the group with diabetes mellitus (8 deaths in 30 days, 19.1%) [**Fig. 4**]. 

**Fig. 5:**
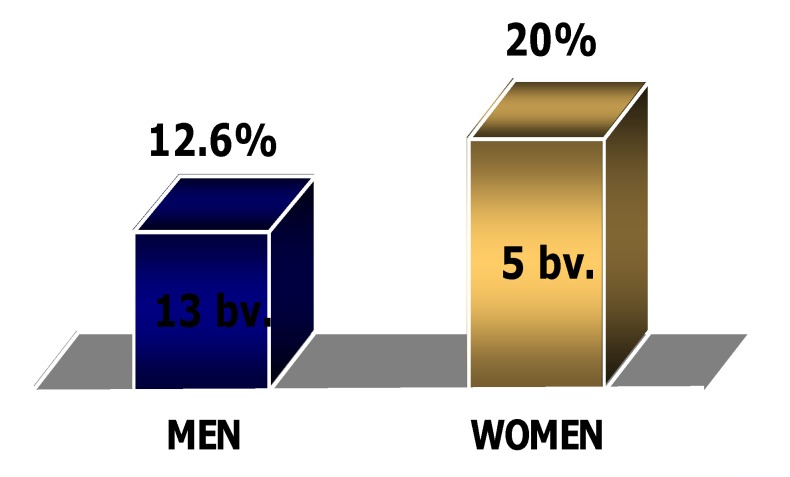
Death rate with respect to sex

The death rate was considerably higher for women than for men (20% vs. 12,6%, p<0,05) – which corresponds to the data in the literature [**Fig. 5**].

**Fig. 6:**
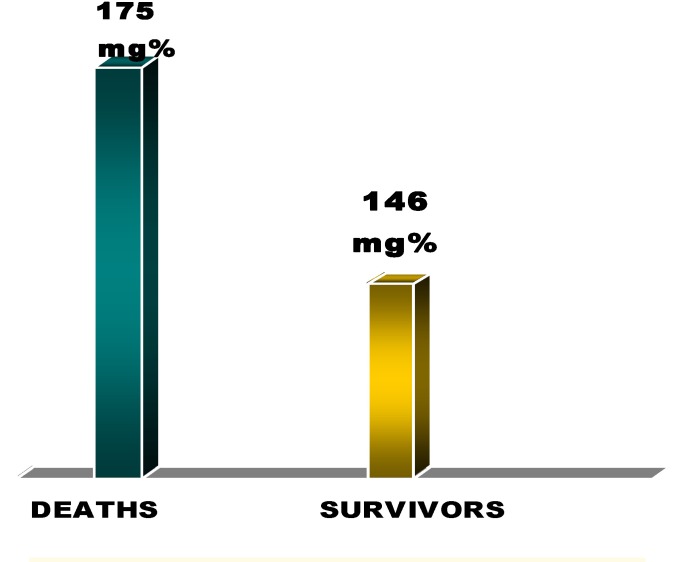
The average blood glucose level in deceased vs. survivors

We independently calculated the mean values of blood glucose level in admittance for the deceased patients and we compared them with the survivors’. The mean value of the initial blood glucose level was higher for the deceased patients than for the survivors – 175± 55mg/dL vs. 146±36mg/dL [**Fig. 6**].

**Fig. 7:**
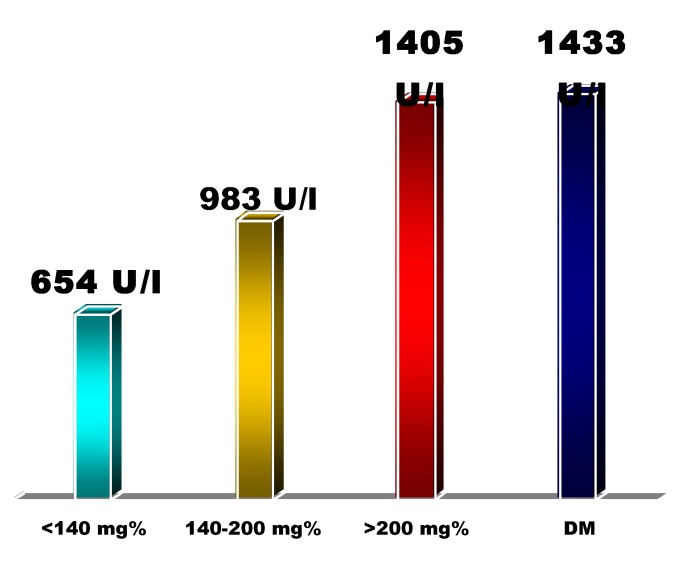
The average level of maximum LDH with respect to the initial glucose blood level

We also found a correlation between the blood glucose level in admittance and the extent of the myocardial infarction (estimated using enzymatic criteria: mean value of maximum LDH), with a significant difference between the extreme groups (blood glucose level<140mg/dL and >200mg/dL, respectively): 654 U/L vs. 1405 U/L (p<0,05) **Fig. 7**.

**Fig. 8:**
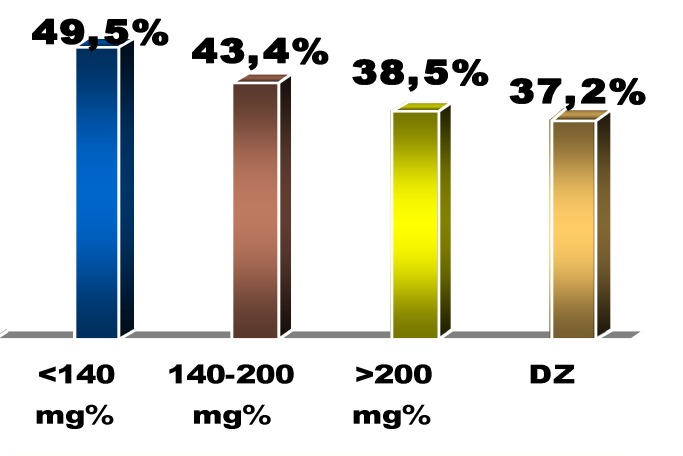
Left ventricle ejection fraction with respect to the initial glucose blood level

By comparison with the echocardiography data (ejection fraction, left ventricle dimensions) in terms of the initial blood glucose level we noticed an inversely proportional correlation between the blood glucose level in admittance and the systolic performance of the left ventricle – characterized by the ejection fraction: 49.5 % (group I) vs. 43.4% (group II) vs. 38.5% (group III) vs. 37.2% (diabetes group), p<0,05 between groups I and III [**Fig. 8**].

We also examined a possible correlation between the initial blood glucose level and the aggravation of the cardiac failure, which was quantified by the increase in NYHA scale, but we found no significant differences:

- in group I – 2 cases, 

- in group II – 3 cases,

- in group III – 2 cases

- in the diabetic patients’ group – 4 cases of progression of the cardiac failure during hospitalization 

 Beyond the clinical evidence of the increased risk of a high blood glucose level in admittance (progressively increasing short-term mortality), we also tried to find whether there is any correlation between the initial blood glucose level and the magnitude of troponin T increase (as well-known biological marker for high risk), but we did not obtain any statistically significant data:

- in group I – the average level of troponin T 0.42ng/mL,

- in group II – the level of troponin T 0.39ng/mL, 

- in group III – troponin T = 0.44ng/mL,

- in the group of diabetic patients – troponinT = 0.48ng/mL.

Troponin T was ingathered for 147 of the 170 patients (115 patients without known diabetes and 32 patients with diabetes). We measured the level of troponin T in admittance, after 8-12 hours, and at 36-48 hours; we chose the maximum value for each patient and obtained a mean value for each group. 

 NTproBNP is another marker with a high predictive value for the rate of complications post myocardial infarction; we measured the level of NTproBNP in 67 patients (out of all 170 monitored in the study) within the first 48 from admittance. 

 We found no significant correlation between the blood glucose level in admittance and the mean value of NTproBNP in the 4 groups of patients:

- group I – NTproBNP = 824pg/mL,

- group II – NTproBNP = 1201pg/mL,

- group III – NTproBNP = 884pg/mL,

- group of patients cu diabetes mellitus - NTproBNP = 2108pg/mL.

We also found no significant difference regarding the rate of mortality with respect to the treatment they received for the initial hyperglycemia: 16 deaths in those treated with insulin (~19%), 8 deaths in those with oral antidiabetics (~21%) and 2 deaths in those who received combined therapy (~22%). 

## DISCUSSION

Approximately a quarter of the patients with myocardial infarction have diabetes mellitus. The rest of 75% represent the majority of patients with acute myocardial infarction out of whom it is estimated that ~ 25% have diabetes that has not been diagnosed yet [**18**], and a large proportion of the others have high initial blood glucose levels, either reversible or not until discharge. These represent a very heterogeneous population at first sight for which the initial blood glucose level can serve as an important element of differentiation regarding the risk stratification.

The negative correlation between diabetes and the prognostic of patients with myocardial infarction can be extended to those with high blood glucose levels in admittance [**19**].

Therefore, a study made in 2002 compares the 1664 consecutive patients with acute myocardial infarction and the evolution of those with diabetes mellitus and of those with high initial blood glucose level >198mg/dL [**20**]; both groups have similar prognostic, significantly more severe than that of normoglycemic one.

Other studies find significant increase in the complications of the interventional procedures made in the myocardial infarction and of the “no reflow” phenomena in those with high blood glucose levels in admittance [**21**]. Others also find a directly proportional correlation between the initial blood glucose level and the blood concentration of CK as an index of myocardial suffering in patients with infarction, which directly contributes to the decrease of the post infarction survival rate [**22**].

A comparison made in 2005 between the blood glucose level in admittance and the *à jeune* blood glucose level within the first 8 hours of hospitalization – as predictor of mortality at 30 days in non diabetic patients with acute myocardial infarction concluded that *à jeune* glycemia is a more accurate marker of a severe evolution [**23**].

It is known that hyperglycemia in admittance, in patients with acute coronary syndromes, increases the in-hospital mortality [**24**], but also that the risk of cardiovascular events is greater in those with pre-diabetic status [**25**].

All these represent enough reasons to look for new solutions for counteracting the serious consequences of the cardiovascular complications in those with dysfunctions of glucidic metabolism:

- repolarising solution (GIK) with contradictory results [**26**,**27**],

- insulin therapy – recommended by the DIGAMI study [**28**,**29**], but less convincing after the DIGAMI2 study [**30**],

- and, especially, the interventional therapies (PCI) and the aggressive antithrombotic ones (GP IIb/IIIa receptor blockers, associated with the dual heparin and antiagregant therapy).

Since other studies have proven the correlation between the glucose blood level in admittance and the rate of complications in patients with acute myocardial infarction with modern reperfusion treatment (including primary angioplasty), this paper wants to show the strong association between the initial glucose blood level (in patients not diagnosed with diabetes mellitus) and short-term mortality (30 days), even in those with acute myocardial infarction without invasive reperfusion treatment. 

In many cases the high blood glucose levels in admittance are a sign of a major metabolic dysfunction (similar to a pre-diabetes status) and are associated to a more severe prognosis. In fact, part of those with high initial glycemia not known as diabetics when admitted, are subsequently diagnosed with impaired glucose tolerance or diabetes mellitus, according to the present diagnosis criteria. 

## Conclusion

Although with some limitations due to the small number of patients (that didn’t allow a complete statistical processing) and the absence of the initial measuring of HbA1C (which would have better appreciated the metabolic profile of the patients), our study proposes a useful and easily available marker (the blood glucose level in admittance) that can contribute to the risk quantification in patients with major coronary syndromes.

The cases with high glycemia in admittance could represent those patients that have an increased response to stress (for instance those with important hemodynamic dysfunction or with a large necrosis mass) and who can develop complications in the ulterior evolution of the myocardial infarction. 

Therefore, we can say that the initial high glycemia seems to be associated with an increased risk in non-diabetic patients and with acute myocardial infarction, probably requiring a more aggressive strategy in order to limit subsequent complications.
